# Admixture and Local Breed Marginalization Threaten Algerian Sheep Diversity

**DOI:** 10.1371/journal.pone.0122667

**Published:** 2015-04-13

**Authors:** Samir Bachir Souheil Gaouar, Anne Da Silva, Elena Ciani, Samia Kdidi, Miloud Aouissat, Laziz Dhimi, Mohamed Lafri, Abderrahman Maftah, Nadhira Mehtar

**Affiliations:** 1 Department of Biology, Aboubakr Belkaid Tlemcen University, Tlemcen, Algeria; 2 Molecular and Cellular Laboratory (USTOM), University of Sciences and Technology, Mohamed Boudiaf, Oran, Algeria; 3 INRA, UMR1061 Génétique Moléculaire Animale, Limoges, France; 4 Université de Limoges, UMR1061 Génétique Moléculaire Animale, Limoges, France; 5 Department of Biosciences, Biotechnologies and Biopharmaceutics, University of Bari, Bari, Italy; 6 Livestock and Wildlife Laboratory, Arid Lands Institute Medenine, Medenine, Tunisia; 7 Laboratory of Genetics, Immunology and Human Pathology, Faculty of Sciences, Tunis-El Manar University, Tunis, Tunisia; 8 Technical Institute of Breeding (ITElv) of Aïn El-Hadjar, Saïda, Algeria; 9 Technical Institute of Breeding (ITElv) of Aïn M’lila, Constantine, Algeria; 10 Laboratoire des Biotechnologies liées à la Reproduction Animale (LBRA) Université de Blida, Blida, Algérie; CSIRO, AUSTRALIA

## Abstract

Due to its geo-climatic conditions, Algeria represents a biodiversity hotspot, with sheep breeds well adapted to a patchwork of extremely heterogeneous harsh habitats. The importance of this peculiar genetic reservoir increases as climate change drives the demand for new adaptations. However, the expansion of a single breed (Ouled-Djellal) which occurred in the last decades has generated a critical situation for the other breeds; some of them are being subjected to uncontrolled cross-breeding with the favored breed and/or to marginalization (effective size contraction). This study investigated genetic diversity within and among six of the nine Algerian breeds, by use of 30 microsatellite markers. Our results showed that, in spite of the census contraction experienced by most of the considered breeds, genetic diversity is still substantial (average gene diversity ranging 0.68 to 0.76) and inbreeding was not identified as a problem. However, two breeds (Rembi and Taâdmit) appeared to have lost most of their genetic originality because of intensive cross-breeding with Ouled-Djellal. Based on the above evidence, we suggest Hamra, Sidaoun, and D’man as breeds deserving the highest priority for conservation in Algeria.

## Introduction

The Near East and North Africa (NENA) region is recognized as the reservoir of genetically unique breeds [1] playing crucial roles in the livelihood of the human populations of this area [2]. Nevertheless, the region is facing continuing erosion of livestock genetic resources, mainly as a consequence of local livestock replacement by more productive breeds, and the generally limited amount of progress made in establishing conservation schemes for threatened breeds. In Algeria, sheep breeding represents 80% of the total domestic animal production (with 18 million head) and mutton provides more than 58% of the national red meat production. Almost all the Algerian ovine livestock belongs today to nine native breeds (Ouled-Djellal, D’man, Hamra, Rembi, Taâdmit, Sidaoun, Tazegzawt, Berbère and Barbarine), strongly adapted to harsh environmental conditions (such as water and/or food scarcity, high temperatures, etc.). Among them, a single breed, Ouled-Djellal, currently accounts for more than 63% of the Algerian sheep population [3]. The breed was introduced by the Romans, wool users [4, 5], in the fifth century from Apulia in Italy. First, the breed was principally reared in the region of Biskra (in the northeastern part of the country); by 1970 its expansion across the country began with facilitated access to motor vehicles. Today, the Ouled-Djellal can be found in most areas of the country excepted in the mountainous and Saharan zones. The increasing farmers’ preference of this breed has been based on a supposedly higher profitability, which has never been scientifically demonstrated outside the breed development area. Uncontrolled cross-breeding with Ouled-Djellal is a relatively frequent practice with some breeds [6]. However, the extent of this phenomenon is still not known due to lack and/or absence of information, which is generally recognized as a main weakness in monitoring local domestic diversity in developing countries [7]. For this reason, we carried out a genetic diversity analysis on six main Algerian sheep breeds using 30 microsatellite markers. Our goal was two-fold: (i) to investigate the level of genetic diversity within breeds, like Hamra, D’man, Taâdmit, and Sidaoun, that are known to have experienced strong population size contraction during the last years, and (ii) to evaluate the possible influence of genetic admixture among breeds on the original genetic make-up of Algerian native breeds.

## Material and Methods

### Ethics Statement

The blood used for all of the analyses was collected by veterinarians during routine blood sampling on commercial farm animals (for medical care or follow up). Those animals were not linked to any experimental design and blood sampling was not performed specifically for this study, therefore no ethical authorization was required. All the samples and data processed in our study were obtained with the breeders and breeding organizations' consent.

### Breeds and samples

We sampled six thin-tailed Algerian breeds, out of which four (Hamra, Rembi, Ouled-Djellal, Taâdmit) fall into the "wool sheep" group, one (D’man) into the “mixed hair-wool sheep” group and one (Sidaoun) into the “hairy sheep” group. The latter, also known as Targui (Targuia, Sidaou or Sidaho) is exploited under nomadic conditions by the Tuareg people in the southern part of Algeria (Central Sahara) [8]. Originated from Mali, it is a highly rustic breed, well adapted to long distance “transhumance” and harsh climatic conditions [9]. The Ouled-Djellal represents the typical sheep breed of steppe and high planes (Central and Eastern Algeria). Animals are characterized by big body size and appreciated for meat production [5, 10]. The Rembi (or Rumbi) is a rustic breed from the Saharan Atlas, well adapted to high-altitude environments, considered by some Authors as a derivative of the so-called “Arab” sheep stock, similarly to Ouled Djellal [4, 5]. The Hamra (also known as Bèni-Ighil, Beni Guil or Deghma), originated from the western plateau, is characterized by a smaller body size and is appreciated for meat organoleptic quality [10, 11]. Crosses between Ouled Djellal and a Merino breed carried out in the second half of the 19^th^ century would have been the basis for the development of the Taâdmit sheep [10]. The D’Man (also known as D’man or Touaregh or Tafilalet) is a small size sheep, originated in the southern and western part of Algeria, raised almost permanently in the oases [9].

Hamra, Sidaoun and D’man look phenotypically well differentiated among each other, while Taâdmit, Ouled-Djellal and Rembi show some phenotypic similarities, such as white fleece; hence, in what follows, we refer to these three breeds as “white breeds”. Minor Algerian breeds, such as the Barbarine, the Tazegzawt and the Berbère breeds were not investigated in this study because of difficulties encountered in the field to access to rare flocks. Indeed, sampling Algerian sheep breeds is not an easy task given their distribution on the territory: most sheep in the north of the country, at a high concentration in the steppe and the semi-arid highlands (75% of the total number), and some breeds even in Saharan regions; which implies the need for a sampling covering almost the whole area of the country (2,381,741 km^2^). Moreover, some breeds (such as D’man and Sidaoun) are located in harsh environments (desert) which also are politically insecure.

Hence, out of a larger sample dataset collected by veterinarians during routine blood sampling on commercial farm animals, carried out in a span of time that goes from 1998 to 2004, we selected for this study a targeted dataset (N = 158) of animals belonging to the six considered breeds. In order to maximize sample representativeness and minimize genetic relationship among individuals, as far as possible, different farms were visited for each breed, and individuals were chosen according to their genealogy. Blood samples were cryopreserved until DNA extraction and analysis.

Details about breed characteristics and sampling are reported in S1 Table, sampling locations are shown in S1 Fig., and pictures of breed specimens are shown in S2 Fig.

Additional data from seven Italian sheep breeds (Bagnolese, Laticauda, Comisana, Sarda, Gentile di Puglia, Altamurana, Leccese), for a total of 739 individuals (for more details, see [12]), were also used as a reference, by the use of the F_ST_ metric. These values, provided calibration points for the interpretation of the genetic division between the Algerian native populations, and were obtained from a set of 15 microsatellites shared between the studies.

### DNA extraction, polymerase chain reaction (PCR) and fragment analysis

Genomic DNA was purified from whole blood by protease K digestion and a salting-out procedure [13]. Thirty-one microsatellites were amplified (S2 Table), out of which 19 were chosen through the panel of microsatellites proposed by the Food and Agriculture Organization of the United Nations/International Society for Animal Genetics (FAO/ISAG) [14]. The adopted markers were distributed across 18 chromosomes, with seven chromosomes harboring two microsatellites each; one chromosome (OAR9) harboring four microsatellites; all the remaining chromosomes harboring a single microsatellite. CSRM60 did not show a satisfying pattern of amplification and was therefore eliminated from the analyses.

For the microsatellites listed in group A: PCR amplification was carried out in 10 μL volumes consisting of 0.2 mM dNTPs, 0.5U to 2.5U QIAGEN HotStar Taq DNA Polymerase, 1.5 mM MgCl_2_, 0.1 μM of each primer and buffer 1X. Amplifications were performed in a GeneAmp PCR System 9600 Thermal Cycler with the following program: 15 min at 95°C; 30 cycles of 30 s at 94°C, 30 s at the annealing temperature, see S2 Table), 30 s at 72°C; and a final extension of 7 min at 60°C. Amplification products were analyzed on non-denaturing polyacrylamide gel electrophoresis (6%) and visualized by silver nitrate staining. Reproducibility was checked through triplicate analysis of all samples and data were interpreted by two independent operators.

For the microsatellites listed in group B: fragment length polymorphism analyses were realized at the “Institut Agro-Vétérinaire Hassan II”, Rabat, Maroc, where operating methods used to be unreported and not disclosed. Annealing temperatures recorded in S2 Table are extracted from literature and are indicative.

For the microsatellites listed in group C: multiplex reactions were carried out using QIAGEN Multiplex PCR Master Mix with fluorescently labeled primers. The 5-plex was composed of: FAM-INRA035, HEX-CSSM66, HEX-BM8125, PET-DYMS1, NED-CSRM60; the 3-plex was composed of: FAM-ETH10, PET-MAF33 and NED-MCM140. PCR amplification of microsatellite loci was carried out in 25 μL volume and included 12.5 μL 2X QIAGEN Multiplex PCR Master-Mix (QIAGEN Multiplex PCR Buffer, containing dNTPs, QIAGEN HotStar Taq DNA Polymerase, 3 mM MgCl_2_, and 0.3 μM of each primer) 1.25 μL of double-distilled water and 4 μL of template DNA (25ng/μL). Amplifications were performed in a GeneAmp PCR System 9600 Thermal Cycler with the following program: 5 min at 95°C; 30 cycles of 30 s at 94°C, 90 s at the annealing temperature (S2 Table), 30 s at 72°C; and a final extension of 30 min at 60°C. Amplification products were loaded on an ABI 3730 Genetic Analyzer using LIZ-600 as internal size standard (Applied Biosystems). Amplified fragment lengths were assigned to allelic sizes with GeneMapper v.4.0 (Applied Biosystems).

Each microsatellite was genotyped across all samples using the same method. Moreover, genotyping methods (A, B, and C) were used in the same way for each Algerian breed considered. Italian breeds were genotyped with another method (for details see [12]) and hence were only used as reference.

### Data Analysis

The mean number of alleles per breed, the average observed (H_o_) and expected (H_e_) heterozygosity over loci per breed were estimated using ARLEQUIN 3.5 [15]. Petit et al. [16] suggested that populations of higher priority for conservation efforts can be determined by considering allelic richness. To calculate allelic richness and the richness of private alleles, we used the rarefaction method [17] implemented in HP-RARE [18] adopting a sample of 16 genes, corresponding to 8 individuals. Polymorphic Information Content (PIC) and effective number of alleles (Na_e_) were estimated for all markers using the Molkin software (version 2.0) [19].

Departures from Hardy–Weinberg equilibrium (HWE) and linkage disequilibrium (LD) among loci were estimated using the program GENEPOP v4.0 [20]. Levels of significance were adjusted using the false discovery rate (FDR) procedure [21].

Some breeds showed an excess of homozygotes (see Results); such excess can be due to nonrandom mating and⁄or the presence of null alleles. Therefore, we used the Expectation-Maximization (EM) algorithm implemented in INEST (http://genetyka.ukw.edu.pl/INEst10_setup.exe) to estimate the frequency of null alleles at each locus and for each breed, in order to take into account simultaneously null allele frequencies at each locus and the average level of the intra-population inbreeding as a multi-locus parameter [22].

The unbiased estimator of Wright inbreeding coefficient, F_IS_, was calculated following Weir and Cockerham [23] (f estimator). Its significance was assessed using a permutation method (10000 permutations) implemented in the GENETIX Version 4.01 package [24].

The extent of population subdivision was examined by calculating the global multi-locus F_ST_ value. The index of pair-wise F_ST_ of Weir and Cockerham [23] and their associated 95% confidence intervals were determined using GDA [25].

A Bayesian model-based clustering approach was used to search for the occurrence of genetic groups (i.e., clusters, K) in our dataset (as implemented in STRUCTURE 2.3.3, [26–29]). The burn-in length of the Markov Chain Monte Carlo (MCMC) was set to 50,000 followed by 200,000 iterations. The admixture model and the correlated allele frequencies model were used without priors on sampling information. Fifteen runs were conducted for each K value, with K ranging from 1 to 6. The most probable value of K was estimated by inspection of ΔK [30] statistics using Structure Harvester [31]). CLUMPP (v. 1.1.1) [32] was used to align the repetitions for each K and the visualization was made by the program DISTRUCT (v.1.1) [33].

To assess the degree to which breeds differ from each other when adopting an approach without assumptions about HWE or LD, we performed Discriminant Analysis of Principal Components (DAPC). A multivariate DAPC analysis performs a preliminary data transformation step using Principal Component Analysis (PCA) to create uncorrelated variables that summarize total variability (e.g., within- and between groups). These variables are then used as input to DA, which aims to maximize between-group variability and achieve the best discrimination of genotypes into predefined clusters. We used the approach implemented in the ADEGENET package [34] within the statistical package R version 3.0.1 [35].

## Results

The thirty microsatellites loci surveyed were all polymorphic; a total of 404 different alleles (mean = 13.6 per locus, s.d. = 4.16) were found in the six breeds, with a mean PIC of 72.24 (s.d. = 15.89). On average, 88% of individuals were successfully typed for each microsatellite (values ranged from 70% for ILSTS5 to 99% for SRCRSP9).

The mean number of alleles ranged from 6.00 (D’man) to 9.13 (Rembi). After adopting the rarefaction procedure, the mean allelic richness ranged from 4.97 (for Hamra) to 6.16 (for Rembi) considering a sample size of 8 individuals (Table 1). We verified that mean allelic richness were not significantly different (Kruskal-Wallis chi-squared = 0.31, df = 2, p-value = 0.85) according to the genotyping method used (A, B or C). Neither distributions of allelic richness nor distribution of private allelic richness were significantly different between breeds (respectively: Kruskal-Wallis chi-squared = 9.40, df = 5, p-value = 0.09; Kruskal-Wallis chi-squared = 9.57, df = 5, p-value = 0.09).

**Table 1:** Genetic diversity measured by breed.

Breed	n	n Loc. Samp.	MNA (s.d.)	R (PR)	Ho (s.d.)	He (s.d.)	Loci not in HWE (FDR*^3^)	F_IS_ IC 95%
Hamra	30	1 (GF*1)	6.83 (2.38)	4.97 (0.38)	0.66 (0.17)	0.70 (0.17)	6 (4)	0.05 [-0.02–0.06]
O.-Djellal	30	1 (GF*1)	8.30 (2.67)	5.84 (0.52)	0.66 (0.20)	0.74 (0.17)	11 (7)	0.11 [0.06–0.12]
Rembi	27	3	9.13 (3.67)	6.16 (0.72)	0.69 (0.18)	0.76 (0.13)	10 (10)	0.11 [0.04–0.13]
Sidaoun	28	1 (RM*2)	7.40 (3.09)	5.35 (0.37)	0.58 (0.19)	0.70 (0.17)	13 (11)	0.18 [0.11–0.21]
Taâdmit	30	1 (GF*1)	8.07 (2.71)	5.66 (0.45)	0.70 (0.15)	0.74 (0.14)	6 (3)	0.05 [0.00–0.06]
D’Man	13	1 (GF*1)	6.00 (2.35)	5.10 (0.39)	0.64 (0.25)	0.68 (0.19)	5 (1)	0.06 [-0.03–0.07]
Mean (s.d.)			7.62 (2.61)	5.51 (1.76)	0.66 (0.18)	0.72 (0.16)		0.09 [0.06–0.13]

n, sample size; n Loc. Samp., number of Locales Sampling; GF*^1^, governmental farms; RM*^2^, Regional market; MNA, Mean Number of Alleles; R, allelic Richness; PR, Private allelic Richness; H_e_, expected heterozygosity; H_o_, observed heterozygosity; HWE, Hardy Weinberg Equilibrium; FDR*^3^, loci in HWD after False Discovery Rate correction [21] and F_IS_, inbreeding coefficient (estimator of Weir and Cockerham [23]).

The observed heterozygosity (H_o_) ranged from 0.58 (Sidaoun) to 0.70 (Taâdmit) with a mean of 0.66 (s.d. = 0.18). H_e_ ranged from 0.68 (D’man) to 0.76 (Rembi) with a mean of 0.72 (s.d. = 0.16) (Table 1). Distributions of H_o_ and H_e_ were not significantly different between breeds (Kruskal-Wallis for H_e_: chi-squared = 7.86, df = 5, p-value = 0.16; Kruskal-Wallis for H_o_: chi-squared = 9.44, df = 5, p-value = 0.09). Calculations with the software INEST suggested possible null alleles at the MCM527 locus (for 20% of individuals) for Sidaoun and at the OARFCB193 locus (for 24% of individuals) for D’man; in the other breeds, the frequency estimated for null alleles never exceeded 0.1. Hence, the explanation of null alleles, on its own, seemed insufficient to explain deviation from Hardy Weinberg Equilibrium. Such deviations were found to be significant, after False Discovery Rate correction, for 7, 10 and 11 loci (out of the 30 considered markers) respectively in Ouled-Djellal, Rembi and Sidaoun. Deviations from HWE were always due to homozygote excess, in agreement with F_IS_ values significantly different from zero for these three breeds (with the highest value for Sidaoun: F_IS_ = 0.18). For the other breeds, the F_IS_ values were positive but not significantly different from zero (Table 1). The mean F_IS_ found was 0.09 [0.06–0.13] IC_95%._

We failed to detect significant linkage disequilibrium between pairs of loci in each considered breed after False Discovery Rate correction [21].

Considering all the breeds, the mean F_ST_ was 0.038 [0.029–0.047] (IC_95%_). Pair-wise F_ST_ values between breeds were particularly low between the Ouled-Djellal/Rembi, Ouled-Djellal/Taâdmit, Rembi/Taâdmit pairs (Table 2). FSTAT [36] was used to calculate and compare mean F_ST_ values among the “white breeds” (formed by Ouled-Djellal/Rembi/Taâdmit) and among the group formed by Sidaoun/D’man/Hamra: mean F_ST_ were respectively of 0.011 and 0.077 and were found significantly different (p-value = 0.050).

**Table 2:** Pair-wise F_ST_ among Algerian breeds (with confidence intervals at 95%).

	Hamra	Ouled-Djellal	Rembi	Sidaoun	Taâdmit
O.-Djellal	0.043 [0.03–0.06]				
Rembi	0.035 [0.024–0.047]	0.009 [0.002–0.017]			
Sidaoun	0.081 [0.055–0.107]	0.030 [0.016–0.044]	0.030 [0.017–0.044]		
Taâdmit	0.039 [0.028–0.052]	0.015 [0.007–0.024]	0.007 [0.002–0.012]	0.034 [0.019–0.051]	
D’man	0.085 [0.054–0.115]	0.052 [0.031–0.075]	0.051 [0.031–0.072]	0.062 [0.041–0.085]	0.053 [0.034–0.072]

As a general reference, mean and pair-wise F_ST_ values were also calculated among seven Italian sheep breeds (Bagnolese, Laticauda, Comisana, Sarda, Gentile di Puglia, Altamurana, Leccese) from a previously published dataset [12], using a set of 15 shared microsatellites loci (Table 3). A higher mean F_ST_ value (0.064, [0.055–0.075] IC_95%_) was found for the Italian breeds compared to the Algerian ones (0.036, [0.026–0.048] IC_95%_). Pair-wise F_ST_ values between Rembi/Taâdmit, Ouled-Djellal/Rembi and Ouled-Djellal/Taâdmit were significantly lower than all the pair-wise F_ST_ values observed between Italian breeds; the only exception concerned the F_ST_ values between Ouled-Djellal/Taâdmit and Bagnolese/Laticauda which showed overlapped confidence intervals. Bagnolese and Laticauda are known to be genetically very close, as they share a common origin and the same breeding area [12].

**Table 3:** Pair-wise F_ST_ among Algerian breeds (a) and Italian breeds (b), calculated with a common set of 15 microsatellites* (with confidence intervals at 95%).

**(a)**	Hamra	Ouled-Djellal	Rembi	Sidaoun	Taâdmit	
O.-Djellal	0.038 [0.020–0.057]					
Rembi	0.037 [0.021–0.052]	0.004 [–0.000–0.013]				
Sidaoun	0.086 [0.057–0.118]	0.032 [0.013–0.053]	0.032 [0.014–0.054]			
Taâdmit	0.040 [0.025–0.053]	0.014 [–0.000–0.026]	0.006 [–0.000–0.012]	0.034 [0.016–0.055]		
D’man	0.082 [0.049–0.123]	0.031 [0.014–0.048]	0.044 [0.019–0.071]	0.055 [0.024–0.087]	0.044 [0.017–0.073]	
**(b)**	Altamurana	Bagnolese	Comisana	Gentile Di Puglia	Laticauda	Leccese
Ba	0.091 [0.065–0.122]					
Co	0.064 [0.040–0.091]	0.060 [0.034–0.091]				
Ge	0.046 [0.032–0.059]	0.066 [0.041–0.089]	0.043 [0.029–0.056]			
La	0.080 [0.045–0.120]	0.024 [0.014–0.032]	0.076 [0.054–0.102]	0.060 [0.037–0.081]		
Le	0.045 [0.030–0.060]	0.069 [0.038–0.104]	0.035 [0.026–0.047]	0.034 [0.026–0.045]	0.081 [0.051–0.120]	
Sa	0.091 [0.071–0.113]	0.108 [0.065–0.164]	0.056 [0.045–0.070]	0.078 [0.057–0.101]	0.119 [0.071–0.181]	0.074 [0.048–0.109]

Ba, Bagnolese; Co, Comisana; Ge, Gentile Di Puglia; La, Laticauda; Le, Leccese; Sa, Sarda.

*the common set of microsatellites was: MAF65, MAF214, OARFCB304, ILST11, OARAE129, OARFCB193, MAF209, OARJMP58, ILST5, OARFCB128, INRA63, BM1824, MAF33, MCM140, BM8125.

**Fig 1:**
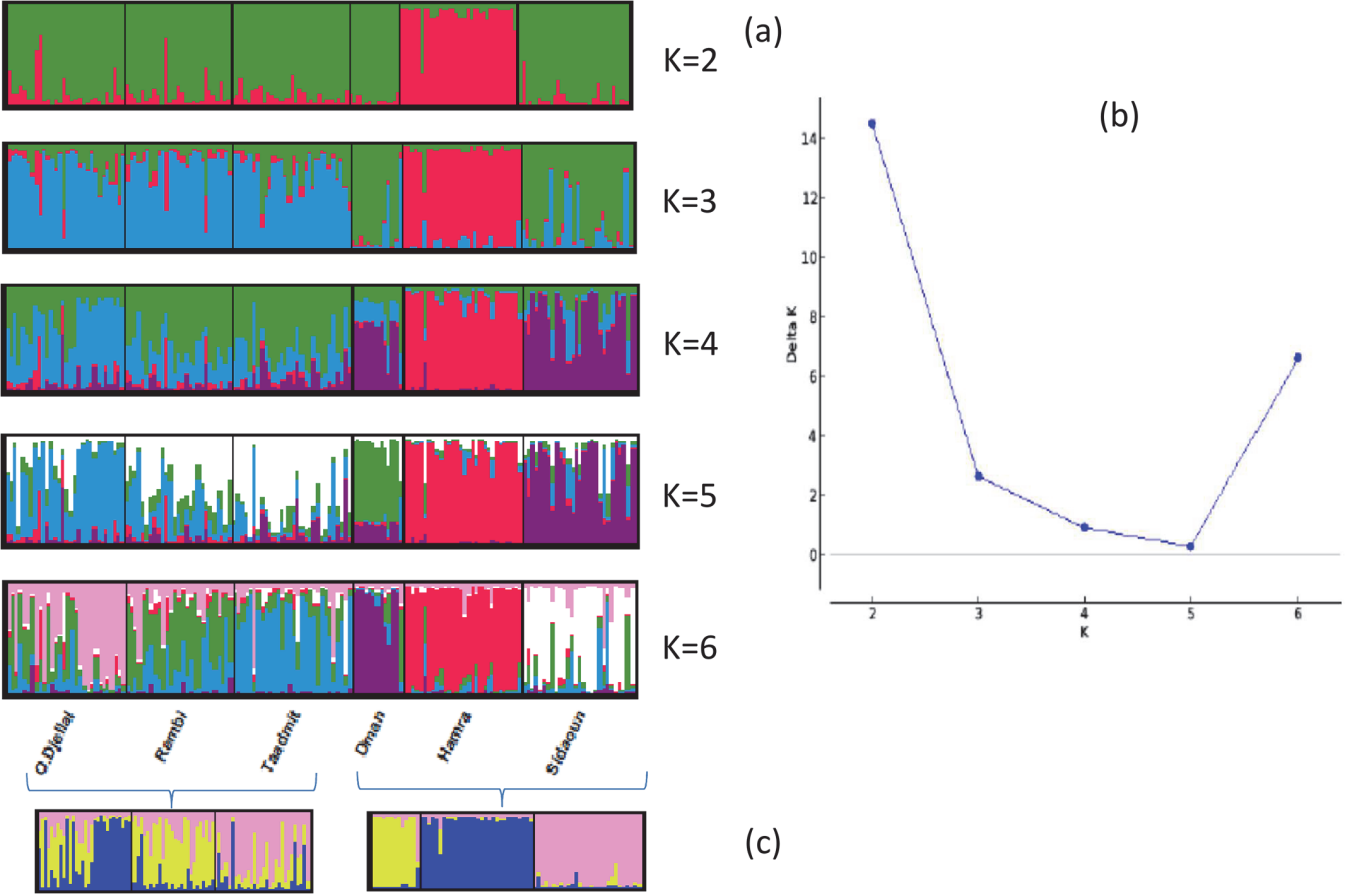
Genetic structure of Algerian sheep breeds by Bayesian analysis with 30 microsatellite loci (K = number of clusters). (a) Analysis on the entire data set with increasing number of inferred clusters (K = 2 to K = 6); (b) Graph showing ΔK calculated according to Evanno et al. [30]; (c) further analysis in the group of “white breeds” (Ouled-Djellal, Rembi and Taâdmit) and the group of the three other breeds (D’man, Hamra and Sidaoun).

The Bayesian analyses for cluster assignment suggested K = 2 as the most likely number of clusters using the ΔK criterion by Evanno et al. [30], with ΔK = 14.49 (Fig. 1A). For K = 2 (Fig. 1A) a clear differentiation between Hamra and the other breeds was evident. A smaller ΔK peak was detected for K = 6 (with ΔK = 6.63) (Fig. 1B), showing that, to a lesser extent, the Bayesian analysis was able to distinguish the 6 nominal breeds. We realized a finer analysis (Fig. 1C) by decomposing our population sample into two subsamples (the “white breeds” and a group including the other three breeds) and performing the Bayesian analysis on the two different subsamples separately. The most likely number of clusters in the “white breeds” was K = 3 (with a ΔK = 6.90). Similarly, in the group including Hamra, D’man and Sidaoun, the best number of clusters was K = 3 with a clearly higher ΔK of 119.83. STRUCURE allowed estimating the proportion of each individual’s genome assigned to each group. The three “white breeds” showed clear admixture (proportion of individual genome assigned to the breed of origin: Ouled-Djellal = 56.5%, Rembi = 64.5%, Taâdmit = 64.2%). On the contrary, almost no admixture was detected for D’man, Hamra, and Sidaoun (with respectively, 88.6%, 95.1%, and 93.5% for the proportions of individual genome assigned to the breed of origin).

**Fig 2:**
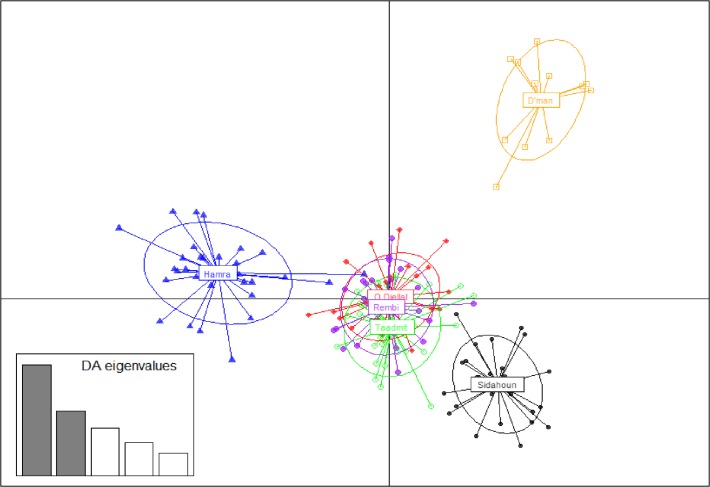
Scatterplot of the first two principal components of DAPC using breeds as prior clusters. Breeds are labeled inside their 95% inertia ellipses and dots represent individuals. The inset indicates the eigenvalues of the first five principal components.

In the DAPC analysis, 80 PCs of the PCA were retained as input to DA, accounting for approximately 89% of the total genetic variability. The scatterplot of the first two components of the DA (Fig. 2) showed that the three “white breeds” were set apart from the three others, which formed a tight cluster with no discernible structure. No clear overlap of the inertia ellipses existed between the “white breeds” and the others (Sidahoun, D’man and Hamra). A high proportion of individuals were correctly assigned to their original group, using the classification functions obtained in the DA, for Sidahoun, D’man and Hamra (respectively: 92.4%, 99.9% and 92.8%), whereas the three “white breeds” were clearly admixed (for Ouled-Djellal, 39.3% of individuals were assigned to Ouled-Djellal, 32.3% to Rembi and 27.8% to Taâdmit; for Rembi 31.6% of individuals were assigned to Rembi, 35.3% to Ouled-Djellal and 32.0% to Taâdmit; for Taâdmit, 39.1% of individuals were assigned to Taâdmit, 28.0% for Ouled-Djellal and 28.7% to Rembi).

## Discussion

The current study investigated the genetic diversity of six main Algerian breeds, enabling the acquisition of original information concerning the level of variability, both within and among breeds.

### Genetic diversity and inbreeding

Some of the breeds studied have been largely marginalized over time, implying reduced flock size, mostly because of farmers' preference for the Ouled-Djellal. Hamra, Sidaoun, Taâdmit and D’man show flock contraction, and contribute currently to a very low proportion of the Algerian sheep livestock (DAGRIS: Domestic Animal Genetic Resources Information System, www.dagris.ilri.cgiar.org; FAO DAD-IS database: www.fao.org/dad-is). If reductions in flock size experienced by breeds are correlated with reductions in effective population size low genetic diversity could be found for these breeds.

Consideration of the six breeds showed moderately high genetic diversity, with homogenous levels of expected heterozygosity, allelic richness and private allelic richness among the breeds. When comparing Algerian breeds with Italian breeds, using a set of fifteen common microsatellites, gene diversity in Algerian breeds was significantly (p-values<0.05), but to a moderate extent, lower (mean H_e_ = 0.72, mean AR = 5.29) than that observed in Italian breeds (mean H_e_ = 0.77, mean AR = 7.54). Hence, in spite of flock size reductions experienced by Hamra, Sidaoun, D’man and Taâdmit, the genetic diversity appeared moderately high for these breeds and in any case not significantly lower than the genetic diversity of Ouled-Djellal. This conclusion must be considered critically, as blood collection goes back to the period 1998–2004. Over recent years the situation has gradually deteriorated [6]; as a consequence, the genetic diversity of these breeds may be currently lower.

Population contraction affects genetic diversity in two ways: (i) some alleles or allele combinations can be lost from the population; (ii) the limited animal numbers in a breed can imply an increased inbreeding level. The mean F_IS_ found in the study was 0.09 [0.06–0.13] IC_95%_. Three breeds, Ouled-Djellal, Rembi and Sidaoun, showed more than 23% of the loci in Hardy-Weinberg disequilibrium with values of F_IS_ close to 0.1, implying heterozygote deficit. According to our analysis, the presence of null alleles could not, on its own, explain these results. Other studies have also reported heterozygote deficit in domestic sheep (see, for example, [37–41]). For Rembi and Sidaoun, these results could most likely be due to subdivision among flocks (Wahlund effect) (see [42] for an analysis of the effect of subdivision in sheep), as for these two breeds the sampling strategy implied visits in different farms which could have contributed to this apparent heterozygote deficit. Ouled-Djellal, sampled on a single flock, also showed positive F_IS_ that hence cannot be explained by a Wahlund effect. This value of F_IS_ could result in reproduction mismanagement in the pilot farm sampled, with an insufficient number of rams used.

In sum and according to our sampling, inbreeding did not appear as an immediate problem for the breeds considered.

### Genetic dilution

Cross-breeding between low and high productive breeds aims to improve breeds faster than through selection schemes, but such practices do not always achieve the desired results [43]. This practice is one of the major threats leading to the disappearance of local genetic diversity, inducing genetic erosion by dilution or eradication of the local genetic pool [44].

In order to evaluate the level of genetic dilution in Algerian breeds due to uncontrolled cross-breeding with the pervasive Ouled-Djellal breed, pair-wise F_ST_ values were calculated. Very low F_ST_ values (ranging 0.007 to 0.015) were observed between breeds belonging to the "white breeds" group while pair-wise F_ST_ values between the remaining breeds were significantly higher. Pair-wise F_ST_ values were also calculated between seven Italian breeds, using a common set of 15 microsatellites, in order to provide a general reference term to interpret pair-wise F_ST_ values between Algerian breeds. The comparison clearly showed poor genetic differentiation among the “white breeds”, with pair-wise F_ST_ values significantly lower than those observed for Italian breeds known to be genetically differentiated. Considering the known origin of Taâdmit and the hypothesized origin of Rembi, both as Ouled-Djellal-derived breeds, limited F_ST_ values were expected in this group; however such very low values of F_ST_ are likely the consequence of cross-breeding phenomena largely recorded between Ouled-Djellal and the two other breeds [6].

The Bayesian analysis showed signals of admixture between the “white breeds” whereas Hamra, Sidaoun and D’man proved to be well differentiated. In spite of this observed levels of admixture, the model-based clustering algorithm implemented in the STRUCTURE software was able to distinguish Ouled-Djellal, Rembi and Taâdmit when they were analyzed together in a reduced dataset. Results of DAPC were more telling, highlighting such a low differentiation level between the three “white breeds” that they appeared almost completely overlapping; while, Hamra, Sidaoun and D’man clearly differentiated from each other and from the central cluster formed by the “white breeds”.

Hence, we can suppose that cross-breeding has spread to such a degree in Algeria that Rembi and Taâdmit prove to be genetically very close to Ouled-Djellal. For Rembi, practices of cross-breeding with the popular Ouled-Djellal led to the disappearance of the initial specimen’s phenotype (characterized by uni-coloured bay-fawn fleece, as described by Chellig [5]), replaced by individuals with uniform white fleece [10]. This phenotypic evolution presaged what is now confirmed by this study at the genetic level. Similarly, the genetic originality of the Taâdmit could have been lost likely due to uncontrolled cross-breeding with Ouled-Djellal. On the contrary, the picture for Hamra, D’man and Sidaoun is reassuring in terms of genetic dilution, as the three breeds appeared preserved from cross-breeding with Ouled-Djellal. For Sidaoun, this statement is rather logical; indeed this breed is highly adapted to Saharan conditions (resistance to conditions of water scarcity and hot temperatures) and so it represents the choice breed for Tuaregs [11]. Moreover, governmental directives prohibit traffic of Sidaoun specimens out of the Saharan area, in order to limit transmission of viruses specific to desert zones. Hence gene flows are quite limited between Sidaoun and any other breed [9].

This study investigates the genetic diversity within and among Algerian breeds. Previous studies on Algerian sheep used very limited number of microsatellites and/or focused only on phylogenetic purposes [45–47]. Our results showed that Rembi and Taâdmit have lost their genetic originality to such an extent that it is now difficult to differentiate subjects from these breeds from animals belonging to the Ouled-Djellal breed. Hamra, Sidaoun and D’man, on the contrary, were not affected by the admixture phenomenon, and furthermore they showed no deficit in genetic diversity. These breeds are highly adapted to harsh environments. They show genetic traits that are or may be critical [11], including those that affect disease resistance and environmental tolerance and therefore they should be given priority for conservation.

## Supporting Information

S1 Table Breed details for the six Algerian sheep breeds; phenotypic description, geographic localization, demographic status, adaptive traits, and sample information. (TIF)

S2 Table Primer sequences, annealing temperature, chromosomal location, and references of the used microsatellite markers. Microsatellites are grouped (group A, B or C) according to amplification and fragment analysis conditions (see details in Material and Methods, section 2). Chr, Chromosomal location; Ref, References; Ta, annealing Temperature; Na_e_, effective number of alleles.
(PDF)

S3 Table The data file consists of microsatellite allele lengths for 30 loci described in the paper. The data file has individuals organized in rows, with the name of the population in the first column. The subsequent columns correspond to microsatellite data (two columns per locus). Missing data are coded by zeros.
(CSV)

S1 Fig Geographical location of the sampling sites for the six Algerian breeds. Hamra, lattitude: 34° 45' 34.06"N longitude: 0° 8' 44.51"E; Rembi, lattitude: 35° 13' 12.84"N longitude: 2° 19' 2.04"E; Ouled-Djellal, latitude: 36°17'N longitude: 6°37'E; Sidaoun, latitude: 22°47'N longitude: 5°31'E; Taâdmit, latitude: 34° 43'N longitude: 3° 13'E; D’man, latitude: 31°37'N longitude: 2°14' W.
(TIF)

S2 Fig Photography of rams for each considered Algerian breed. On the top, the three “white breeds” (from the left to the right: Ouled-Djellal—Taâdmit—Rembi); on the bottom, from the left to the right: Sidaoun—D’Man—Hamra.
(TIF)
