# Supplementary figures and images for: Admixture and Local Breed Marginalization Threaten Algerian Sheep Diversity
Source: PLoS One. 2015 Apr 13;10(4):e0122667. doi: 10.1371/journal.pone.0122667 (PMC4395297; doi:10.1371/journal.pone.0122667)

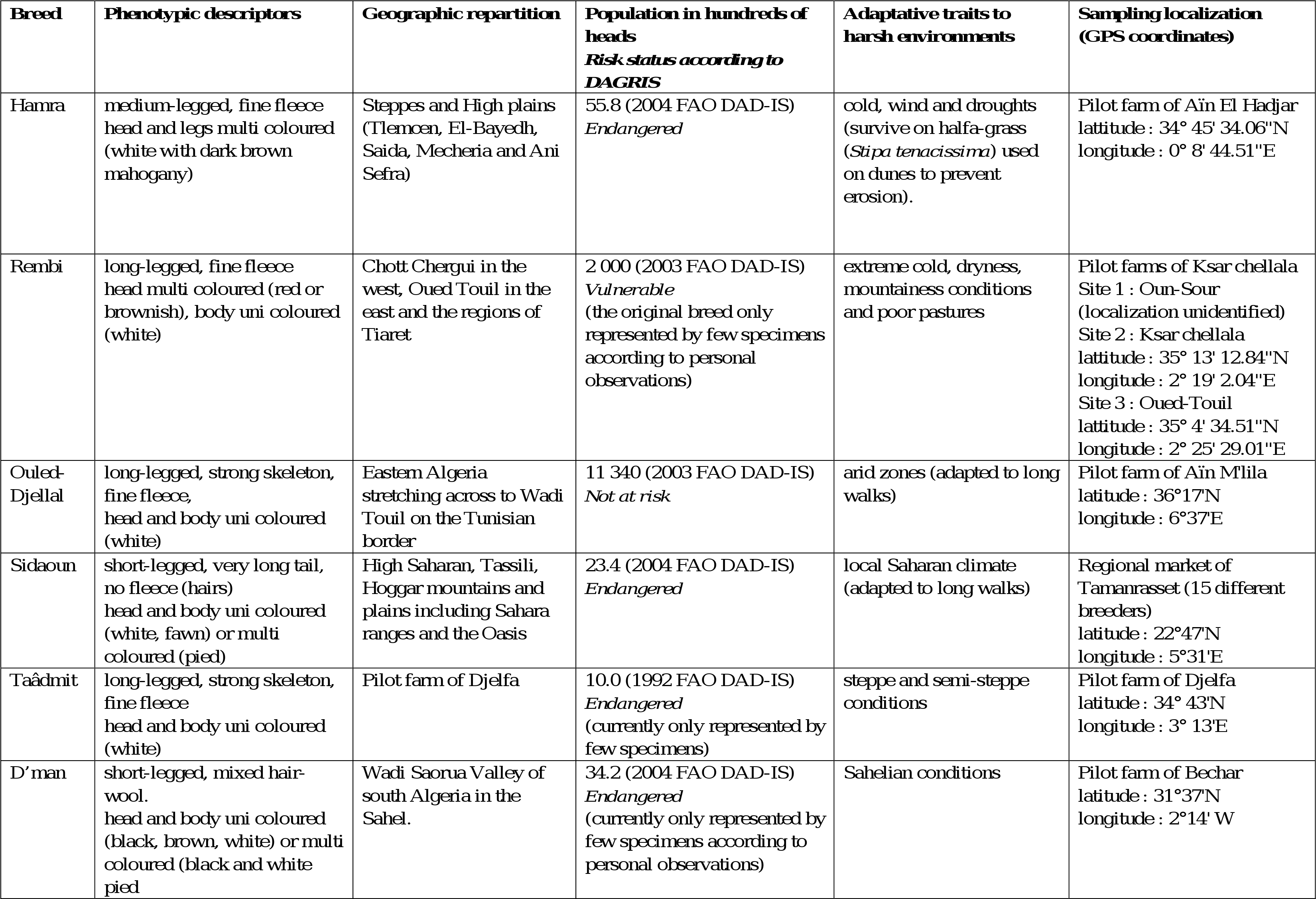

Supplement: S1 Table — (TIF) [file pone.0122667.s001.tif]

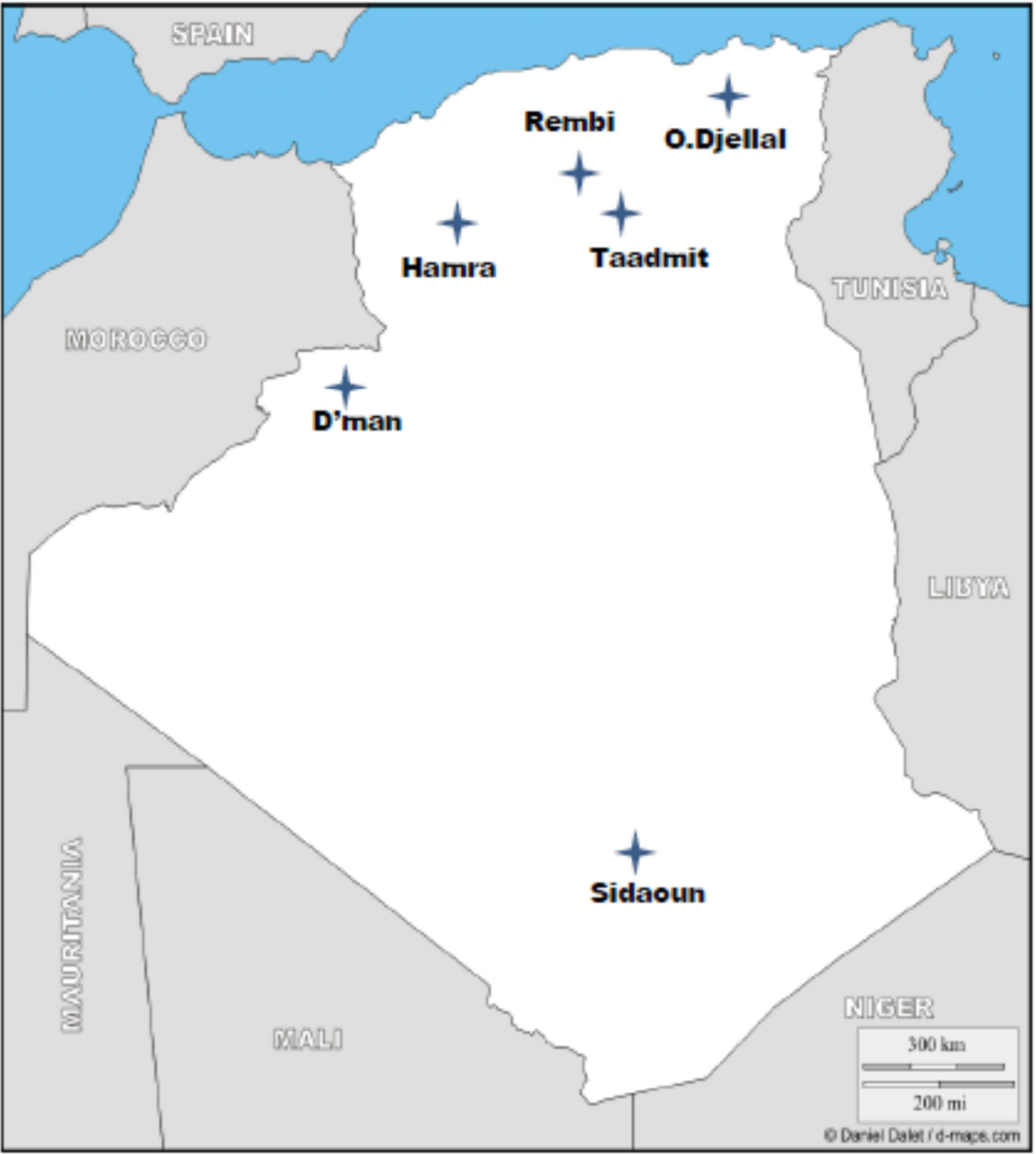

Supplement: S1 Fig — Hamra, lattitude: 34° 45' 34.06"N longitude: 0° 8' 44.51"E; Rembi, lattitude: 35° 13' 12.84"N longitude: 2° 19' 2.04"E; Ouled-Djellal, latitude: 36°17'N longitude: 6°37'E; Sidaoun, latitude: 22°47'N longitude: 5°31'E; Taâdmit, latitude: 34° 43'N longitude: 3° 13'E; D’man, latitude: 31°37'N longitude: 2°14' W. (TIF) [file pone.0122667.s004.tif]

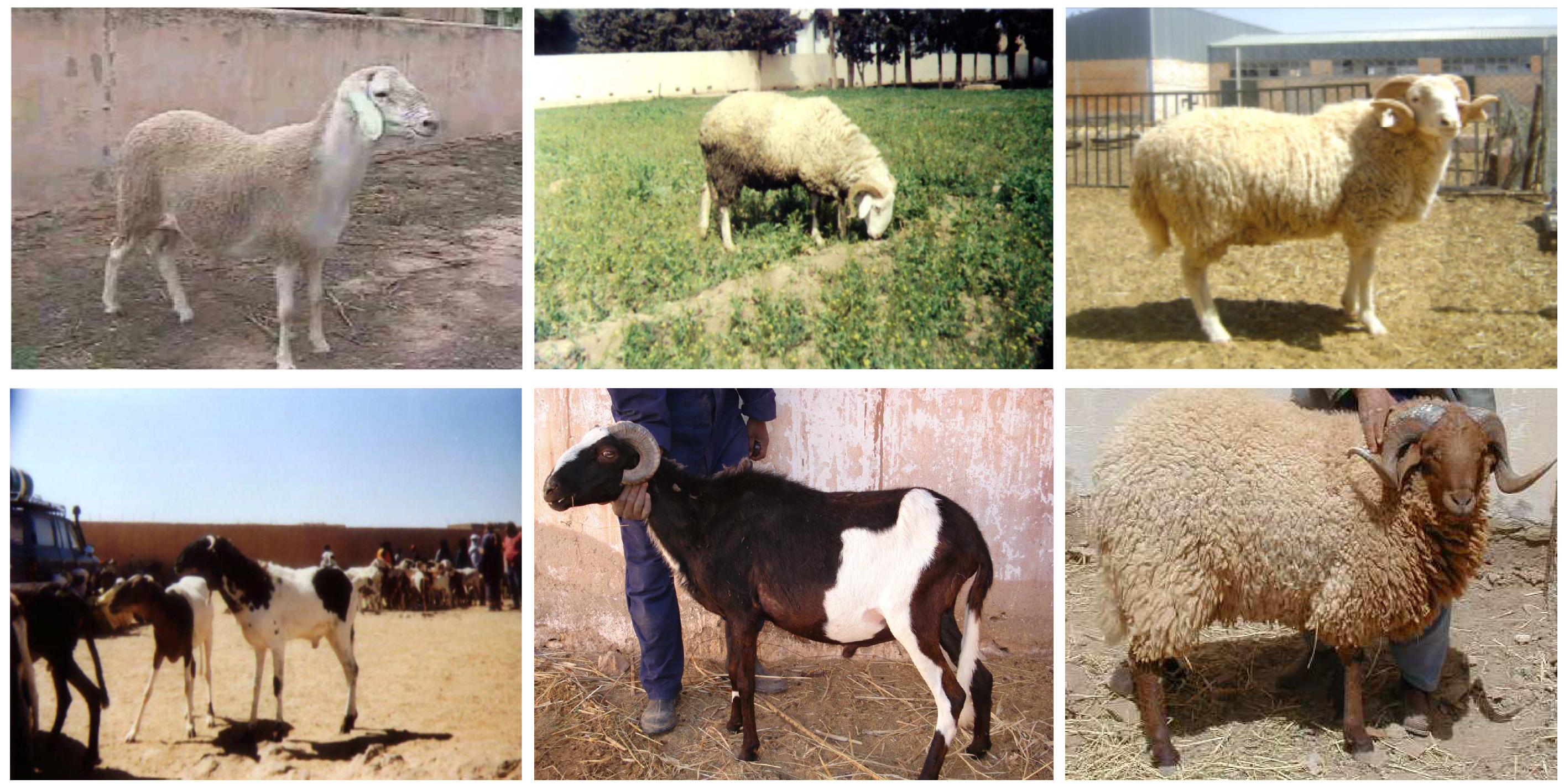

Supplement: S2 Fig — On the top, the three “white breeds” (from the left to the right: Ouled-Djellal—Taâdmit—Rembi); on the bottom, from the left to the right: Sidaoun—D’Man—Hamra. (TIF) [file pone.0122667.s005.tif]
