# Supplementary material for: Admixture and Local Breed Marginalization Threaten Algerian Sheep Diversity
Source: PLoS One. 2015 Apr 13;10(4):e0122667. doi: 10.1371/journal.pone.0122667 (PMC4395297; doi:10.1371/journal.pone.0122667)
Supplement: S2 Table — Microsatellites are grouped (group A, B or C) according to amplification and fragment analysis conditions (see details in Material and Methods, section 2). Chr, Chromosomal location; Ref, References; Ta, annealing Temperature; Nae, effective number of alleles. (PDF) [file pone.0122667.s002.pdf]

Locus Name (Group)	Chr	Primers sequences	Genebank accession number	Ref	Ta	Na_e
OarFCB128 (A)	2	FOR: 5’ATTAAAGCATCTTCTCTTTATTTCCTCGC-3’<br>REV: 5’- CAGCTGAGCAACTAAGACATACATGCG-3’	L01532	[1]	64°C	6.2
OarCP34 (A)	3	FOR: 5’- GCTGAACAATGTGATATGTTCAGG-3’<br>REV: 5’- GGGACAATACTGTCTTAGATGCTGC-3’	U15699	[2]	62°C	3.8
INRA063 (A)	14	FOR: 5’- ATTTGCACAAGCTAAATCTAACC-3’<br>REV: 5’- AAACCACAGAAATGCTTGGAAG-3’	X71507	[3]	55°C	7.8
BM1824 (A)	1	FOR: 5’-GAGCAAGGTGTTTTTCCAATC-3’<br>REV: 5’-CATTCTCCAACTGCTTCCTTG-3’	G18394	[4]	57°C	3.3
OarFCB193 (B)	11	FOR: 5’- TTCATCTCAGACTGGGATTCAGAAAGGC-3’<br>REV: 5’-GCTTGGAAATAACCCTCCTGCATCCC-3’	L01533	[1]	54°C	8.8
OarFCB304 (B)	19	FOR: 5’- CCCTAGGAGCTTTCAATAAAGAATCGG-3’<br>REV: 5’-CGCTGCTGTCAACTGGGTCAGGG-3’	L01535	[1]	56°C	5.7
INRA 49 (B)	1	FOR: 5’-TGTATTAGTTTGTGTTCTTTGGC-3’<br>REV: 5’-TTGGCTTCCACAATCACACA-3’	X71588	[3]	52°C	2.8
MCM 527 (B)	5	FOR: 5’-GTCCATTGCCTCAAATCAATTC-3’<br>REV: 5’-AAACCACTTGACTACTCCCCAA-3’	L34277	[5]	50°C	5.1
MCM42 (B)	9	FOR: 5’- CATCTTTCAAAAGAACTCCGAAAGTG-3’<br>REV: 5’-CTTGGAATCCTTCCTAACTTTCGG-3’	L34281	[5]	55°C	2.9
MAF65 (B)	15	FOR: 5’- AAAGGCCAGAGTATGCAATTAGGAG-3’<br>REV: 5’- CCACTCCTCCTGAGAATATAACATG-3’	M67437	[6]	60°C	4.3
TGLA 53 (B)	12	FOR: 5’-CAGCAGACAGCTGCAAGAGTTAGC-3’<br>REV: 5’- CTTTCAGAAATAGTTTGCATTCATGCAG-3’	-	[7]	52°C	8.6
OarFCB20 (B)	2	FOR: 5’-AAATGTGTTTAAGATTCCATACAGTG-3’<br>REV: 5’-GGAAAACCCCCATATATACCTATA C-3’	L20004	[8]	62°C	10.2
CSRD 247 (B)	14	FOR: 5’-GGACTTGCCAGACTCTGCAAT-3’<br>REV: 5’- CACTGTGGTTTGCATTAGTCAGG-3’	EU009450	[9]	55°C	5.4
HSC (B)	20	FOR: 5’- CTGCCAATGCAGAGACACAAGA-3’<br>REV: 5’-GTCTGTCTCCTGTCTTGTCATC-3’	M90759	[10]	65°C	10.6
SRCRSP9 (B)	CHI12 (goat)	FOR: 5’-AGAGGATCTGGAAATGGAATC-3’<br>REV: 5’-GCACTCTTTTCAGCCCTAATG-3’	L22201	[11]	55°C	3.5
HUJ616 (B)	13	FOR: 5’-TTCAAACTACACATTGACAGGG-3’<br>REV: 5’-GGACCTTTGGCAATGGAAGG-3’	M88250	[12]	54°C	4.2
ILSTS11 (B)	9	FOR: 5’-GCTTGCTACATGGAAAGTGC-3’<br>REV: 5’-CTAAAATGCAGAGCCCTACC-3’	L23485	[13]	55°C	4.2
OarAE129 (B)	5	FOR: 5’-AATCCAGTGTGTGAAAGACTAATCCAG-3’<br>REV: 5’- GTAGATCAAGATATAGAATATTTTTCAACACC-3’	L11051	[14]	54°C	2.7
MAF209 (B)	17	FOR: 5’- GATCACAAAAAGTTGGATACAACCGTGG-3’<br>REV: 5’- TCATGCACTTAAGTATGTAGGATGCTG-3’	M80358	[15]	63°C	2.6
MAF214 (B)		FOR: 5’- GGGTGATCTTAGGGAGGTTTTGGAGG-3’<br>REV: 5’- AATGCAGGAGATCTGAGGCAGGGACG-3’	M88160	[16]	58°C	2.9
OARJMP58 (B)	26	FOR: 5’- GAAGTCATTGAGGGGTCGCTAACC-3’<br>REV: 5’- CTTCATGTTCACAGGACTTTCTCTG-3’	U35058	[17]	52°C	5.7
ILSTS5 (B)	7	FOR: 5’- GGAAGCAATGAAATCTATAGCC-3’<br>REV: 5’-TGTTCTGTGAGTTTGTAAGC-3’	L23481	[18]	55°C	3.5
INRA035 (C)	12	FOR: 5’-ATCCTTTGCAGCCTCCACATTG-3’<br>REV: 5’-TTGTGCTTTATGACACTATCCG-3’	X68049	[3]	57°C	4.8
ETH10 (C)	5 (cattle)	FOR: 5’- GTTCAGGACTGGCCCTGCTAACA-3’<br>REV: 5’-CCTCCAGCCCACTTTCTCTTCTC-3’	Z22739	[19]	60°C	1.6
TGLA122 (C)	18	FOR: 5’-CCCTCCTCCAGGTAAATCAGC-3’<br>REV: 5’- AATCACATGGCAAATAAGTACATAC-3’	-	[20]	60°C	4.9
CSSM66 (C)	9	FOR: 5’- ACACAAATCCTTTCTGCCAGCTGA-3’<br>REV: 5’- AATTTAATGCACTGAGGAGCTTGG-3’	-	[21]	57°C	6.4
BM8125 (C)	17	FOR: 5’-CTCTATCTGTGGAAAAGGTGGG-3’<br>REV: 5’-GGGGGTTAGACTTCAACATACG-3’	G18475	[4]	57°C	2.6
DYMS1 (C)	20	FOR: 5’-AACAACATCAAACAGTAAGAG-3’<br>REV: 5’-CATAGTAACAGATCTTCCTACA-3’	AJ621046	[22]	57°C	7.8
MAF33 (C)	9	FOR: 5’-GATCTTTGTTTCAATCTATTCCAATTTC-3’<br>REV: 5’-GATCATCTGAGTGTGAGTATATACAG-3’	M77200	[23]	60°C	4.4
MCM140 (C)	6	FOR: 5’- GTTCGTACTTCTGGGTACTGGTCTC-3’<br>REV: 5’-GTCCATGGATTTGCAGAGTCAG-3’	L38979	[24]	60°C	4.9
CSRM60 (C)	10 (cattle)	FOR: 5’-AAGATGTGATCCAAGAGAGAGGCA-3’<br>REV: 5’-AGGACCAGATCGTGAAAGGCATAG-3’	-	[25]	57°C	1

1. Buchanan FC, Crawford AM. Ovine microsatellites at the OarFCB11, OarFCB128, OarFCB193, OarFCB266 and OarFCB304 loci. Anim Genet. 1993; 24: 145.
2. Ede AJ, Pierson CA, Crawford AM. Ovine microsatellites at the OarCP34, OarCP38, OarCP43, OarCP49, OarCP73, OarCP79 and OarCP99 loci. Anim Genet. 1995; 26: 130–131.
3. Vaiman D, Mercier D, Moazami–Goudarzi K, Eggen A, Ciampolini R, Lépingle A, et al. A set of 99 cattle microsatellites: characterization, synteny mapping, and polymorphism. Mamm Genome. 1994; 5: 288–297.
4. Bishop MD, Kappes SM, Keele JW, Stone RT, Sunden SL, Hawkins SL, et al. A genetic linkage map for cattle. Genetics. 1994; 136: 619–639.
5. Hulme DJ, Silk JP, Redwin JM, Barendse W, Beh KJ. Ten polymorphic ovine microsatellites. Anim Genet. 1994; 25: 434–435.
6. Buchanan FC, Swarbrick PA, Crawford AM. Ovine dinucleotide repeat polymorphism at the MAF65 locus. Anim Genet. 1992; 23: 85.
7. Georges M, Massey J. Polymorphic DNA markers in Bovidae (World Intellectual Property Org Geneva). WO Publ 92/13102; 1992.
8. Buchanan FC, Galloway SM, Crawford AM. Ovine microsatellites at the OarFCB5, OarFCB19, OarFCB20, OarFCB48, OarFCB129 and OarFCB226 loci. Anim Genet. 1994; 25: 60.
9. Davies KP, Maddox JF, Harrison B, Drinkwater R. Ovine dinucleotide repeat polymorphism at eight anonymous loci. Anim Genet. 1996; 27: 381–382.
10. Scott PC, Maddox JF, Gogolin–Ewens KJ, Brandon MR. The nucleotide sequence and evolution of ovine MHC class II B genes: DQB and DRB. Immunogenetics. 1992; 35: 217.
11. Bhebhe E, Kogi J, Holder DA, Arevalo E, Derr JN, Linn RA, et al. Caprine microsatellite dinucleotide repeat polymorphisms at the SR–CRSP–6, SR–CRSP–7, SR–CRSP–8, SR–CRSP–9 and SR–CRSP–10 loci. Anim Genet. 1994; 25: 203.
12. Shalom A, Soller M, Friedmann A. Dinucleotide repeat polymorphism at the bovine HUJ616 locus. Anim Genet. 1993; 24: 327.
13. Brezinsky L, Kemp SJ, Teale AJ. Five polymorphic bovine microsatellites (ILSTS010–014). Anim Genet. 1993; 24: 75–76.
14. Penty JM, Henry HM, Ede AJ, Crawford AM. Ovine microsatellites at the OarAE16, OarAE54, OarAE57, OarAE119 and OarAE129 loci. Anim Genet. 1993; 24: 219.
15. Buchanan FC, Crawford AM. Ovine dinucleotide repeat polymorphism at the MAF209 locus. Anim Genet. 1992; 23: 183.
16. Buchanan FC, Crawford AM. Ovine dinucleotide repeat polymorphism at the MAF214 locus. Anim Genet. 1992; 23: 394.
17. Lumsden JM, Lord EA, Montgomery GW. Characterization and linkage mapping of ten sheep microsatellite markers derived from a sheep x hamster cell hybrid. Anim Genet. 1996; 27: 203–206.
18. Brezinsky L, Kemp SJ, Teale AJ. ILSTS005: a polymorphic bovine microsatellite. Anim Genet. 1993; 24: 73.
19. Toldo SS, Fries R, Steffen P, Neibergs HL, Barendse W, Womack JE, et al. Physically mapped, cosmid–derived microsatellite markers as anchor loci on bovine chromosomes. Mamm Genome. 1993; 4: 720–727.
20. Georges M, Massey J. Polymorphic DNA markers in Bovidae. Patent WO 92/13102; 1992.
21. Arora R, Lakhchaura BD, Prosad RB, Chauhan A, Bais RKS, Tantia MS, Vijh RK. Physical and microsatellite based characterization of Tarai Buffalo and of India Buffalo. Newsletter. 2003; 19.
22. Buitkamp J, Filmether P, Stear MJ, Epplen JT. Class I and class II major histocompatibility complex alleles are associated with faecal egg counts following natural, predominantly Ostertagia circumcincta infection. Parasitol Res. 1996; 82: 693–696.
23. Buchanan FC, Crawford AM. Ovine dinucleotide repeat polymorphism at the MAF33 locus. Anim Genet. 1992; 23: 186.
24. Hulme DJ, Smith AJ, Silk JP, Redwin JM, Beh KJ. Polymorphic sheep microsatellites at the McM2, McM131, McM135, McM136, McM140, McM200, McM214, McM373, McM505, McM507 and McM512 loci. Anim Genet. 1995; 26: 369–370.
25. Moore SS, Byrne K, Berger KT, Barendse W, McCarthy F, Womack JE, et al. Characterization of 65 bovine microsatellites. Mamm Genome. 1994; 5: 84–90.
